# The Control and Comprehensive Safety Assessment of Heavy Metal Impurities (As, Pb, and Cd) in Green Tea *Camellia sinensis* (L.) Samples (Infusions) Available in Poland

**DOI:** 10.1007/s12011-023-03665-5

**Published:** 2023-05-02

**Authors:** Kamil Jurowski, Elżbieta Kondratowicz-Pietruszka, Mirosław Krośniak

**Affiliations:** 1 Laboratory of Innovative Toxicological Research and Analyses, Institute of Medical Studies, Medical College, Rzeszów University, Al. Mjr. W. Kopisto 2a, 35-959 Rzeszow, Poland; 2 Department of Regulatory and Forensic Toxicology, Institute of Medical Expertises, Aleksandrowska 67/93, 91-205 Lodz, Poland; 3 Department of General Chemistry, Cracow University of Economics, Sienkiewicza 5, 30-033 Krakow, Poland; 4 Department of Food Chemistry and Nutrition, Medical College, Jagiellonian University, Medyczna 9, 30-688 Krakow, Poland

**Keywords:** Green tea, Tea infusions, Toxicological risk assessment (TRA), Heavy metals, ICP-MS

## Abstract

Tea is the world’s most common drink after water, and the world’s annual sales exceed $43 billion, of which more than $11 billion is green tea (*Camellia sinensis* (L.)). Increasing evidence continues to show that green tea consumption and its ingredients have a variety of potential health benefits. The aim of the study was to control and assess the toxicological risk (TRA) of three heavy metals (As, Pb, and Cd) in infusions of green tea (*n* = 12) from Polish markets. We applied the method of inductive coupled plasma mass spectrometry (ICP-MS) to determine the selected heavy metal and designed a specific toxicological risk assessment with three crucial tiers. The first tier of our strategy was to determine the elements investigated (heavy metal impurities, µg/L). The second step was to estimate the weekly intake of green tea infusions (µg/week) based on weekly consumption. The third tier was to estimate the weekly intake per weight (µg/L/week/bw), based on the average weekly intake of green tea infusion per adult compared to the provisional weekly intake (PTWI) established by the FAO/WHO Joint Food Additives Expert Committee (JECFA). The levels of the investigated heavy metals occur at different levels in all of the investigated green tea infusions. The heavy metal profile indicated that As (0.0721–10.585 µg/L), Pb (0.386–1.695 µg/L), and Cd (0.126–0.346 µg/L) were present in all samples. Basic analysis of general content shows that As (average = 0.325 µg/L) was at a level similar to Cd (average = 0.214 µg/L). Surprisingly, the Pb content (average 0.891 µg/L) was approximately 2.75–4 times higher than that of As and Cd, respectively. The application of our proposed safety assessment methodology (three tiers) provides satisfactory results for regulatory purposes. The evaluation of the investigated heavy metals in all products analysed from green tea samples (infusions) showed that there were no health hazards to consumers due to weekly exposure. The results indicated that after drinking green tea infusions from Polish markets, the amount of investigated elements in weekly doses does not represent any health risks to consumers.

**Supplementary Information:**

The online version contains supplementary material available at 10.1007/s12011-023-03665-5.

## Introduction

In general, tea is a popular beverage that is consumed by people all over the world. It contains polysaccharides, caffeine, polyphenols, amino acids, and antioxidants, and is beneficial to human health. Tea is the most widely consumed beverage in the world, with annual sales exceeding $43 billion worldwide, of which more than $11 billion is green tea (*Camellia sinensis* (L.) [4]. Furthermore, evidence is continuing to be produced that green tea consumption and its constituents have potential health benefits [3, 11, 16–19]. The average daily consumption of green tea was three cups for tea consumers [7, 16, 17], while in some countries, it could reach 10 cups per day [11]. However, it should be noted that beer manufacturing practices and cup sizes are not universal. The composition of tea and tea beverages with polyphenols varies greatly depending on the techniques used and the preparations used [9, 10]. In Japan and China, loose tea leaves and tea bags are usually used for tea brewing in hot 100–150 mL cups, and in the USA, tea drinkers use 2.25 g (1 tea bag) in hot 180–240 mL cups [28].

In addition to food, beverages such as green tea can also be important sources of food intake of toxic elements in daily life. Therefore, regular consumption of green tea can contribute to daily nutritional requirements for several elements. Heavy metal impurities (HMI) are particularly important pollutants in the overall environment. HMI is a health hazard and a major public health concern worldwide [2, 12]. However, it is a very rare topic. In Poland, for example, Pb and Cd in diets (850 daily food rations of students) were determined among students from three universities in Lublin (in south-east Poland) to assess the levels of exposure to these contaminants, compared to PTWI and TDI values. Data show that in none of these cases, the intake level reached 70% of the PTWI/TDI value, and therefore, the risk of diseases associated with high exposure to these toxic metals absorbed from food is low. Koch et al. [18] also described a similar study on the diet intake of toxic heavy metals with major groups of food products (different foodstuffs). The results obtained show that both genders may have a high risk of nephrotoxicity due to Pb food intake. The food intake of other elements (Cd, Hg, Ni) was well below the limits set by the EFSA [5]. On the other hand, application of PCA (principal component analysis) showed that cereals and vegetables were major contributors to a total intake of Cd, Ni, and Hg, while water and beverages were major dietary sources of Pb. Why HMI are so important from biological and environmental point of view? HMI are one of the most hazardous contaminants due to its nondegradable, persistent, accumulative, and toxic nature in the environment [20-24]. Important links in the transfer of HMI from the soil to the human body are plants (including herbs used as tea infusions). The level of HMI in herbs is conditional, and the content is influenced by the geochemical properties of the soil and the ability of the herbs to selectively accumulate HMI [20, 23, 24, 26]. Normally, As (it is metalloid, however usually classified in toxicology as heavy metal), Pb, and Cd are classified as HMI in toxicology [24, 27]. It should be noted that because HMI is diversified in soil bioavailability, its content in raw herbs can vary considerably [1, 9, 26, 27]. Therefore, a very important problem in modern environmental analysis is the monitoring and toxicological risk assessment of HMI in consumer products with herbs (Jurowski et al., 2021, [21]. This problem is very important,however, there is a lack of adequate literature on the toxicological analysis of green tea infusion in relation to As, Pb, and Cd.

**Fig. 1:**
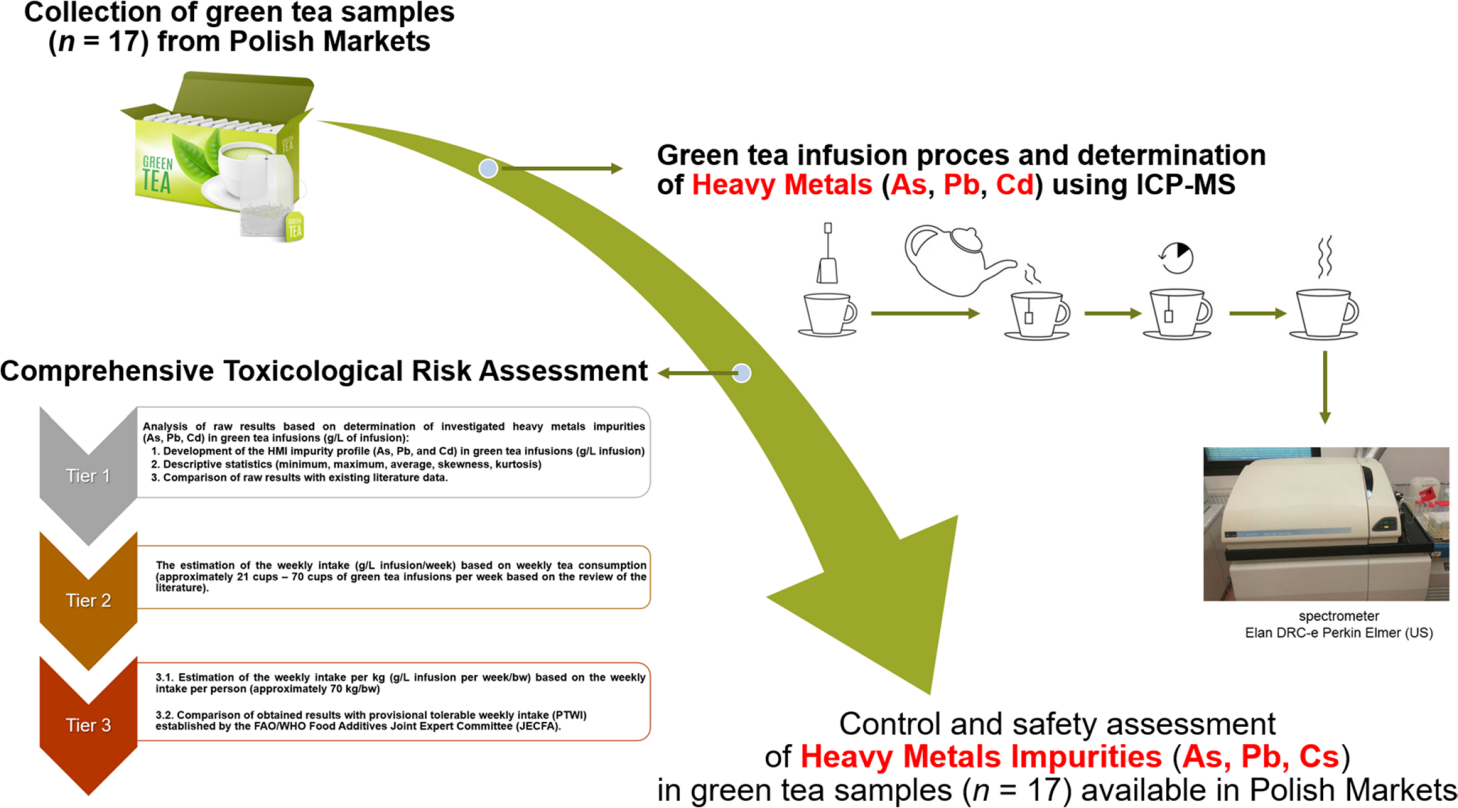
The workflow of the investigated studies: the comprehensive assessment of the toxicological risk of HMI in the green tea infusions available in Poland (*n* = 12)

Therefore, the objective of this study is not to determine the selected heavy metals (As, Pb, Pb, and Cd), but rather to perform a comprehensive toxicological risk assessment of these HMI in green tea infusions available in Polish markets (*n* = 12). The reason for selecting these three metals is that As, Pb, and impurities are (1) the most important in toxicology (heavy metals), (2) our analytical possibilities, and (3) our scientific experience. Therefore, the rarity of this type of research in the field of comprehensive toxicological risk assessment of HMI in consumer products requires appropriate scientific studies. The first tier of our research was analysis of raw results to prepare the HMI impurity profile (As, Pb, and Cd) in green tea infusions (g/L infusion), descriptive statistics, and comparison of raw results with existing data. The second tier was the estimation of the weekly intake (g/L infusion/week) based on weekly tea consumption (approximately 21 cups–70 cups of green tea infusions per week based on the review of the literature [7, 11, 16, 17]. The last tier was the estimation of the weekly intake per kg (g/L infusion per week/bw) based on the weekly intake per person (approximately 70 kg/bw), compared to the provisional tolerable weekly intake (PTWI) established by the FAO/WHO Food Additives Joint Expert Committee (JECFA). The novelty and great advantage of our study is the alternative solution to solve this problem by applying our comprehensive toxicological health risk assessment strategy (three tiers) in contrast to routine approaches (like THQ hazard quotient). The idea of our study is presented schematically as the workflow in Fig. 1.

### Characteristics of Green Tea Samples and Their Collection

Samples of commonly consumed green tea (all available products in Poland; *n* = 12 samples) were randomly collected from general stores in five cities in Poland. Gdańsk (18° 40′E, 54°, 21′N), Kraków (19°56′ E, 50°04′ N), Rzeszów (22° 01 ′E, 50° 03′ N), Poznań (16° 58′E, 52° 25′N), and Warsaw (21°00′ E, 52°15′ N) from 10 July to 19 October 2022. Samples from at least three different series were bought and mixed to make a representative sub-sample. Green tea samples were available on the market in different forms (such as silk, cotton, nylon, paper tea bags, leafy, and needle-like). Most green tea bags were packaged in boxes of 20–25 tea bags (containing 1.4–2.0 g of raw materials) or as raw materials (leaf/ needle). All samples were applied according to the manufacturer’s instructions, without any additional preparation steps (dry, cut, or (pre)wash). The descriptive characteristics of all green tea samples (*n* = 12) were randomly coded as GT1, GT2, etc. All important information about the investigated samples (form, quantity of raw materials for the infusion process, time for the infusion process/brewing process, country of origin, EAN-European identification number) were briefly summarised in the Supplementary Materials SM1 and also in our recently published articles about other elemental impurities in green tea samples [6]

### Chemicals

All chemicals used were of analytical grade and were applied for the preparation of all solutions of demineralised water (Millipore). Ultrapure, demineralised water was obtained by the Milli-Q water purification system (Milipore, Bedford, MA). Nitric acid (65%) was spectral grade (Merck SupraPur, Darmstadt, Germany).Three heavy metals, that is As, Pb, and Cd, were analysed. For this purpose, two multielement stock solutions (CHECL01.13632.0100 and Merck 1.10580.0100) containing As, Cd, and Pb were used as internal standards. The concentrations of elements in multi-element stock solutions are shown in Table 1.

**Table 1:** The level of the analysed elements in applied multi-element stock solution

Element	Concentration in applied multi-element stock solution, mg/L
As	97.0
Pb	9.9
Cd	10.0

### Instrumentations

For simultaneous multi-element detection of As, Pb, and Cd, the ICP-MS technique was applied based on our previous study [14, 15, 21]. The applied analytical instrument was an Elan DRC-e Perkin Elmer (US). Plasma excitation power was 1150 W,the gas flow rates for plasma gas, carrier gas, and makeup gas were 15.0, 1.1, and 1.0 L min^−1^, respectively. The optimised experimental parameters are summarised in Table 2. All details of the analytical calibration strategy and quality control were described in Supplementary Material 2 (SM 2).

**Table 2:** The optimized operating conditions of applied ICP-MS apparatus

Parameter	Value (s)
Instrument	Elan DRC-e Perkin Elmer (US)
Calibration	External
RF power	1150
Dwell Time	250 ms
Sweeps/readings	4
Readings/replicates	2
Replicates	3
Spray chamber	Cyclonic spray chamber
Nebulizer	Meinhard nebulizer
Cooling gas flow rate (L/min)	17
Sampler cone	Ni
Scanning mode	Peak hopping
Plasma gas flow rate	15 L/min
Carrier gas flow rate	1.1 L/min
Composition gas flow rate	1.0 L/min

### The Green Tea Infusion Process Procedure

The green tea infusion process was carried out according to the manufacturer’s requirements described in more detail in Supplementary Materials SM1 (i.e., the amount of raw material and the time of infusion). This process involves adding 200 mL of boiling water to the corresponding amount of tea material (silk/cotton/nylon/paper tea bags or leaves/needle-like) in a 250-mL plastic flask. The tea infusion was mixed with a plastic stirring rod to ensure adequate irrigation and then covered for 3 to 8 min (depending on the recommended time for the boiling of the tea, that is, 3 to 10 min). During the brewing process, we did not add lemon juice or citric acid, as has been done in other studies for black teas [13], because the habits and practises of drinking green tea with added lemon juice or citric acid are not known in the literature. Furthermore, we did not determine the elements of the dry tea, since from a toxicological point of view, the amount of HMI only in the final infusions is important. After infusion, the obtained solution was decanted and cooled to room temperature until analysis was performed using an ICP-MS method. The summary of the idea of our study is schematically summarised as the workflow in Fig. 1.

### Toxicological Risk Assessment Strategy

Our toxicological risk assessment was a comprehensive approach to get a suitable estimate of the health risks of the investigated HMI (As, Pb, and Cd) in the green tea infusions available in Poland (*n* = 17), which consisted of three crucial levels described in Table 3, and presented schematically in Fig. 1.

**Table 3:** Description of the applied triple-tier toxicological risk assessment strategy

Tier	Description
1	Critical analysis of raw results based on determination of investigated heavy metal impurities (As, Pb, Cd) in green tea infusions (g/L of infusion): Development of the HMI impurity profile (As, Pb, and Cd) in green tea infusions (g/L infusion) Descriptive statistics (minimum, maximum, average, skewness, kurtosis); Comparison of raw results with data from the existing literature [8]
2	Estimation of weekly intake (g/L infusion/week) based on weekly tea consumption (approximately 21 cups–70 cups of green tea infusions per week based on the review of the literature [7, 11, 16, 17]
3	3.1. Estimation of the weekly intake per kg (g/L infusion per week/bw) based on the weekly intake per person (approximately 70 kg/bw) 3.2. Comparison of the results obtained with the provisional tolerable weekly intake (PTWI) established by the FAO / WHO Joint Expert Committee (JECFA)

### Statistical Analysis

Preliminary data collection was performed using Excel 2010 (Microsoft Office, authorised by the University of Rzeszów). All data are expressed as five independent replicas of the standard error (average standard error). Statistical descriptive statistics (minimum, maximum, average) were generated using Origin Pro 2022 Pro (licenced by the Jagiellonian University). The elemental impurity profiles were plotted using Origin Pro 2022 (licenced by the Jagiellonian University).

## Results and Discussion

### Tier 1: The Critical Analysis of Raw Results Based on Determination of Investigated Heavy Metal Impurities (As, Pb, Cd) in Green Tea Infusions

The first level of our toxicological risk assessment consists of three important issues (Table 3): (1) development of the HMI impurity profile (As, Pb, and Cd) in green tea infusions (µg/L infusion), (2) descriptive statistics (minimum, maximum, average, skewness, kurtosis), and (3) comparison of raw results with existing literature data [8].

**Fig. 2:**
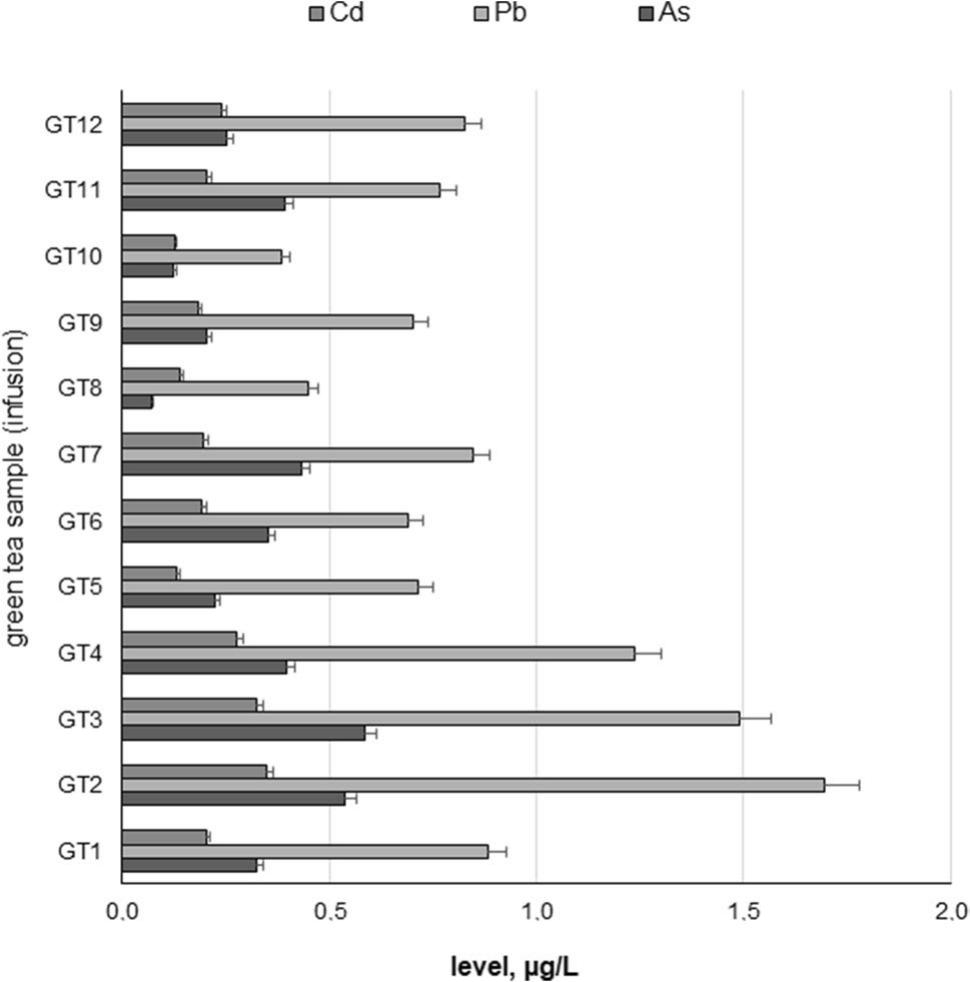
The HMI profile (As, Pb, and Cd) profile of all investigated green tea samples (*n* = 12; GT1–GT12) from Polish markets after the infusion process (µg/L)

**Fig. 3:**
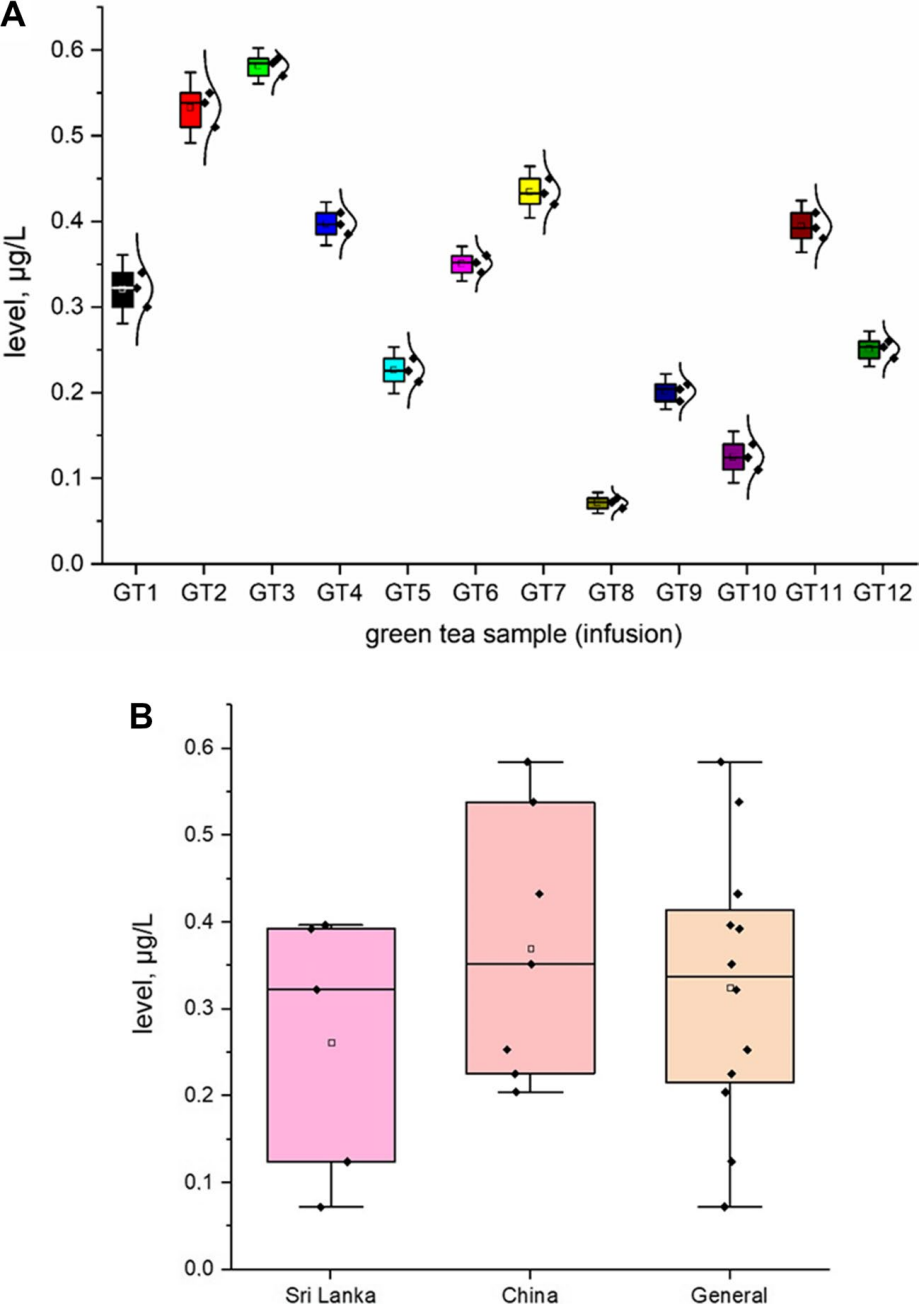
The plot as a box chart with normal distribution curve for the As level (μg/L) in the green tea samples analysed (infusions; GT1–GT12): **A** impurity profile of all samples; **B** impurity profile depending on country of origin

**Fig. 4:**
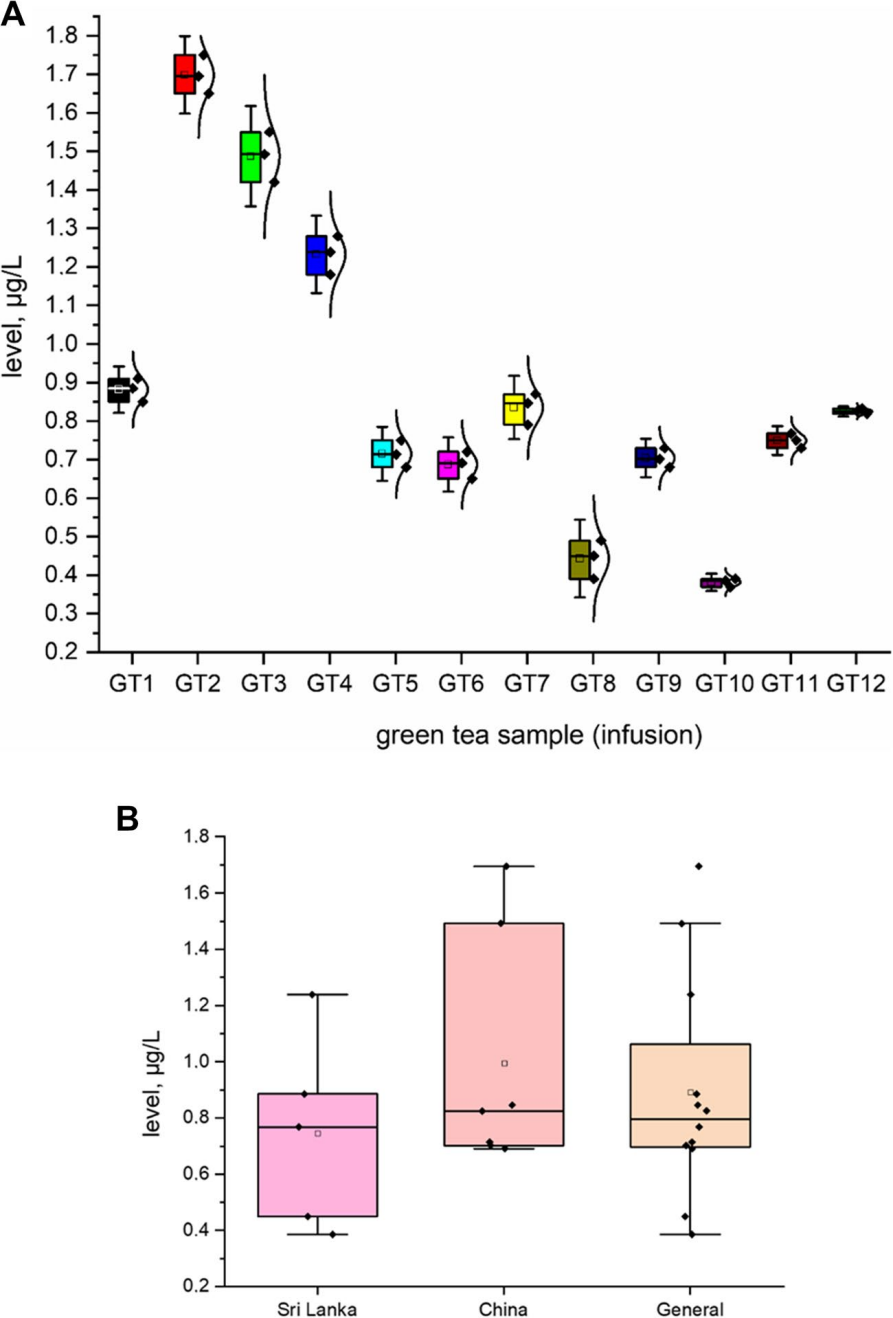
The plot as box chart with normal distribution curve for Pb level (μg/L) in analysed green tea samples (infusions; GT1–GT12)

**Fig. 5:**
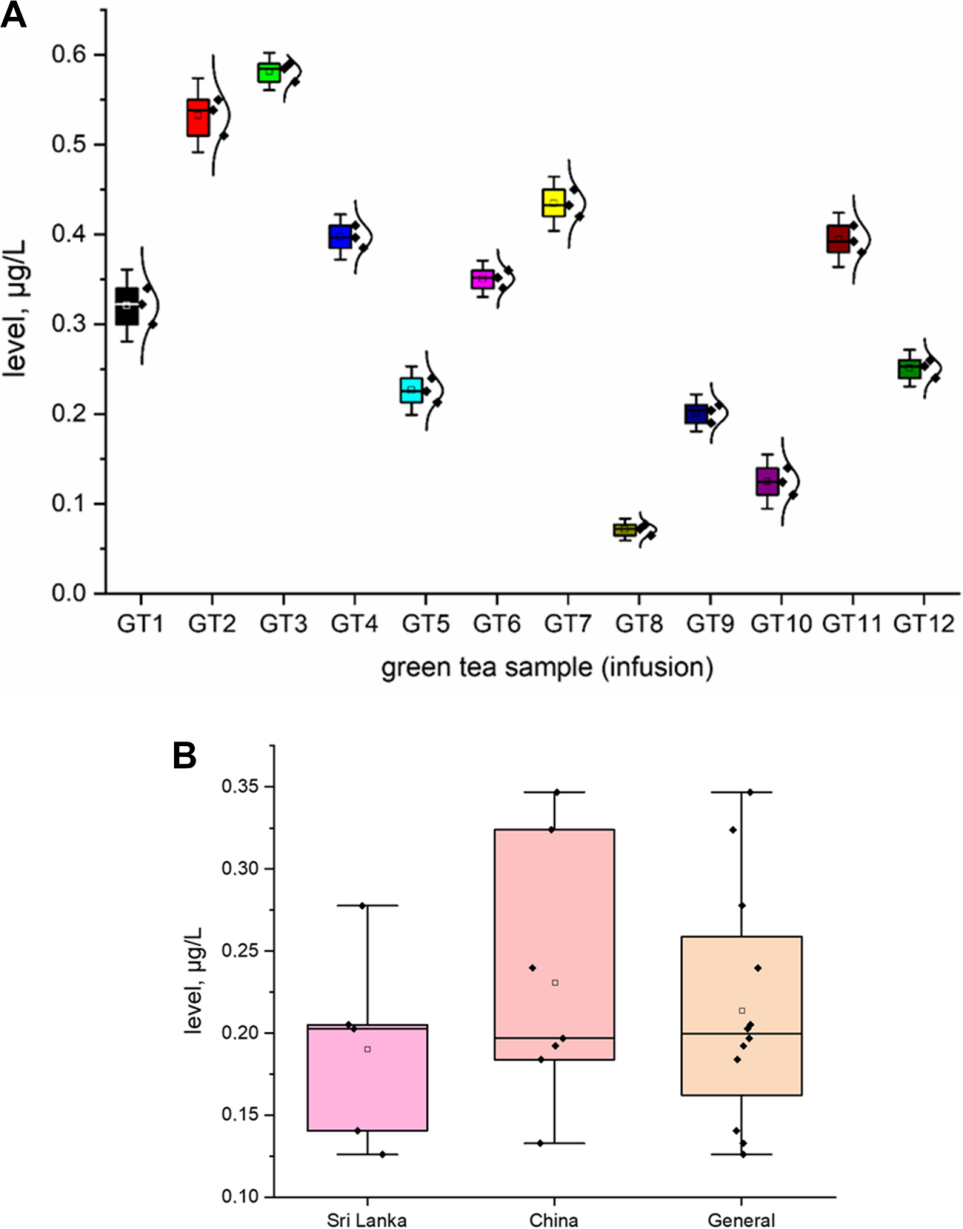
The plot as box chart with normal distribution curve for the level of Cd level (μg/L) in the analysed green tea samples (infusions; GT1–GT12)

All investigated HMI (As, Pb, and Cd; *n* = 12; GT1–GT12) are shown in Fig. 2 as a heavy metal impurity profile. To ensure better transparency and a more accurate analysis of each HMI separately, a box chart with normal distribution curve was prepared (Figs. 3, 4 and 5), including all samples investigated (A) and depending on the country of origin (B). Additionally, descriptive statistics (minimum, maximum, mean, RSD, curve, and skew) are shown in Table 4.

**Table 4:** The descriptive statistics of the investigated HMI in all the analysed samples (GT1–GT12)

Heavy metal	Minimum, μg/L	Maximum, μg/L	Mean, μg/L	RSD, %	Kurtosis	Skewness
As	0.0721	0.585	0.325	0.71–5.5	− 0.64	0.0551
Pb	0.386	1.695	0.891	1.32–8.22	0.35	0.967
Cd	0.126	0.346	0.214	1.91–4.58	− 0.34	0.687

In general, all investigated HMI were present in all green tea samples (infusions) in the range: 0.0721–1.695 μg/L. The As content varies (from 0.0721 to 0.585 μg/L, average = 0.325 μg/L). Individual analysis showed that the lowest As content was observed in GT8 (0.585 ± 0.018 μg/L) and the highest level was in GT3 (0.585 ± 0.09 μg/L). On the other hand, the level of Cd impurities also varies (in the range of 0.126 μg/L to 0.346 μg/L; mean = 0.891 μg/L). Finally, the content of Pb was relatively high (from 0.386 to 1.695 μg/L, average = 0.89 μg/L). Basic analysis of the general content shows that As (average = 0.325 µg/L) was at a level similar to Cd (average = 0.214 µg/L). Surprisingly, the content of Pb (average 0.891 µg/L) was approximately 2.75–4 times higher than that of As and Cd, respectively. In all cases, the level of HMI was higher in samples from China than in Sri Lanka. Skewness and kurtosis values (Table 2) confirm the distribution and consistency of the results obtained.

Only one article is related to the determination of selected elements (As, Pb, and Cd) in green tea infusions [8]. The comparison of the obtained (experimental) values as means with the data from the literature [8] is presented for the HMI investigated in Table 5.

**Table 5:** Comparison of the results obtained (means; µg/L) with values in the literature values (from [8]) for As, Pb, and Cd in green tea infusions

HMI	Mean value in green tea samples (infusion)	
	Experimental mean value, µg/L	Literature value [8], µg/L
As	0.325 ± 0.071	0.09–0.28
Pb	0.891 ± 0.083	0.52–1.31
Cd	0.214 ± 0.029	0.10–0.22

Comparison of the results obtained (mean, µg/L) and the values from the literature [8],µg/L) indicates that our results are quite similar; however, there is no information on the number of green tea samples investigated in the study mentioned. Hence, our observations are indicative, and it is difficult to draw far-reaching conclusions.

### Tier 2: Estimation of Weekly Exposure Based on the Intake of Green Tea

The second level of our toxicological risk assessment of HMI was the estimation of the weekly exposure based on the weekly intake of green tea. This is not easy because there are many ways to apply the analysed product according to the frequency of use. Based on the information mentioned in the introduction, the average daily consumption of green tea (infusion) is approximately three cups per person per day, but in some countries, it can reach 10 cups per day. In Japan and China, loose tea leaves and tea bags are usually used for tea preparation using 100–150 mL, and in the USA, tea consumers use approximately 2.25 g (1 tea bag) using 180–240 mL. Therefore, for our study, we estimate weekly intake (µg/L infusion/week) based on weekly tea consumption (approximately 21 cups–70 cups of green tea infusions per week; 200 mL based on the review of the articles mentioned articles [7, 11, 16, 17]. The results obtained are summarised in Table 6. Due to the lack of any toxicological reference values for this estimation, the discussion for this part is not possible; however, this tier is crucial for the last tier, the estimation of weekly intake depending on body weight based on weekly green tea consumption.

**Table 6:** The estimation of the weekly intake of HMI based on the consumption of weekly green tea infusions

Green tea sample (infusion)	Estimation of weekly intake, µg/week		
	As	Pb	Cd
GT1	1.354–4.512	3.717–12.390	0.85–11.91
GT2	2.261–7.538	7.119–23.731	1.46–20.40
GT3	2.456–8.186	6.267–20.890	1.36–19.05
GT4	1.666–5.552	5.201–17.338	1.17–16.33
GT5	0.947–3.157	2.998–9.992	0.56–7.82
GT6	1.478–4.926	2.902–9.672	0.81–11.31
GT7	1.817–6.057	3.553–11.844	0.83–11.58
GT8	0.303–1.009	1.889–6.298	0.59–8.27
GT9	0.858–2.859	2.946–9.820	0.77–10.82
GT10	0.522–1.741	1.621–5.402	0.53–7.42
GT11	1.647–5.492	3.225–10.748	0.86–12.06
GT12	1.064–3.546	3.465–11.550	1.01–14.10

### Tier 3: Estimation of the Weekly HMI Intake in Green Tea Samples (Infusion) Depending on Body Weight

The last level of our toxicological risk assessment was the estimation of the weekly intake based on body weight based on the weekly consumption of green tea. To do this, the weekly intake for each HMI in the investigated samples (Table 6) was calculated by dividing by 70 kg; average adult human weight recommended by EFSA [5]. The results are shown in Table 7.

**Table 7:** The estimation of weekly intake per body weight based on weekly consumption of green tea

Green tea sample (infusion)	Estimation of weekly intake, µg/week/bw		
	As	Pb	Cd
GT1	1.93E − 02–6.45E − 02	5.31E − 02–1.77E − 01	1.22E − 02–4.05E − 02
GT2	3.23E − 02–1.08E − 01	1.02E − 01–3.39E − 01	2.08E − 02–6.94E − 02
GT3	3.51E − 02–1.17E − 01	8.95E − 02–2.98E − 01	1.94E − 02–6.48E − 02
GT4	2.38E − 02–7.93E − 02	7.43E − 02–2.48E − 01	1.67E − 02–5.55E − 02
GT5	1.35E − 02–4.51E − 02	4.28E − 02–1.43E − 01	7.98E − 03–2.66E − 02
GT6	2.11E − 02–7.04E − 02	4.15E − 02–1.38E − 01	1.15E − 02–3.85E − 02
GT7	2.60E − 02–8.65E − 02	5.08E − 02–1.69E − 01	1.18E − 02–3.94E − 02
GT8	4.32E − 03–1.44E − 02	2.70E − 02–9.00E − 02	8.44E − 03–2.81E − 02
GT9	1.23E − 02–4.08E − 02	4.21E − 02–1.40E − 01	1.10E − 02–3.68E − 02
GT10	7.46E − 03–2.49E − 02	2.32E − 02–7.72E − 02	7.57E − 03–2.52E − 02
GT11	2.35E − 02–7.85E − 02	4.61E − 02–1.54E − 01	1.23E − 02–4.10E − 02
GT12	1.52E − 02–5.07E − 02	4.95E − 02–1.65E − 01	1.44E − 02–4.80E − 02

The appropriate toxicological reference value for the final tier of the applied toxicological risk assessment was the comparison of the estimated weight-dependent weekly intake with the provisional tolerable weekly intake (PTWI) values. This is a widely applied parameter to determine the limits of essential nutrients in the diet, published by the FAO/World Health Organisation (JECFA) Expert Committee on Food Additives (JECFA) [5]. The concept of PTWI was established by the JECFA in 1972 as a weekly intake value, usually expressed as µg of pollutant per kg of body weight per week. FAO/WHO Joint Committee of Experts on Food Additives (JECFA) reviews 22 food additives, including HMI: PTWI_As_ = 0.015 mg/kg [25], PTWI_Pb_ = 0.025 mg/kg [25], and PTWI_Cd_ = 7.0 μg/kg [25]. For better readability, we calculated the ratio of the weekly intake values (µg/kg bw/week) to established PTWIs; results are summarised in Supplementary Materials 3 (SM3). The results obtained (see Supplementary Material 3; SM3) show that the weekly exposure of As, Pb, and Cd to PTWI is generally low (in any case, it does not exceed 1%). Therefore, we conclude that each of the products analysed in Polish markets does not pose any health hazards to consumers, including As, Pb, and Cd, at weekly exposures.

## Conclusions

Proper control and comprehensive toxicological risk assessment of heavy metal impurities of green tea (infusions) are a very important topic; however, there is a lack of appropriate scientific articles. Our study includes toxicological risk assessment based on three crucial tiers. The first level of our strategy shows that the level of heavy metals investigated in all green tea samples (infusions) investigated was relatively low. The heavy metal profile indicated that As (0.0721–10.585 µg/L), Pb (0.386–1.695 µg/L), and Cd (0.126–0.346 µg/L) were present in all samples. The basic analysis of the general content shows that As (average = 0.325 µg/L) was at a level similar to that of Cd (average = 0.214 µg/L). Surprisingly, the content of Pb (average 0.891 µg/L) was approximately 2.75–4 times higher than that of As and Cd, respectively. The second level was to estimate the weekly intake of green tea infusions (µg/week) based on the weekly consumption. The third tier was to estimate the weekly intake per weight (µg/L/week/bw), based on the average weekly intake of green tea infusion per adult compared to the provisional weekly intake (PTWI) established by the FAO/WHO Joint Food Additives Expert Committee (JECFA). The levels of the investigated heavy metals occur at different levels in all of the investigated green tea infusions.

The results obtained show that As, Pb, and Cd are generally low in weekly exposure to PTWI (in any case, no more than 1%). Consequently, we concluded that every product analysed does not present any health risk to consumers at the weekly level, including As, Pb, and Cd. The results indicated that the HMI levels in the weekly dose should not represent any health hazard to the consumer after drinking green tea (infusions) available in Polish markets.

To the best of our knowledge, our comprehensive triple-tier HMI research strategy provides pioneering data, which may be valuable to other researchers and manufacturers. In addition, well-designed toxic risk assessment methods are useful and important for regulatory toxicology studies.

### Supplementary Information

Below is the link to the electronic supplementary material.

Supplementary file1 (DOCX 19 KB)

Supplementary file2 (DOCX 15 KB)

Supplementary file3 (DOCX 15 KB)

## Data Availability

All data generated or analysed during this study are included in this published article and its supplementary information file.

## Abbreviations

EAN: European Article Number
HMI: Heavy metal impurities
ICP-MS: Inductively coupled plasma mass spectrometry
JECFA: Joint FAO/WHO Expert Committee on Food Additives
PTWI: Provisional tolerable weekly intake
