# Supplementary material for: The Control and Comprehensive Safety Assessment of Heavy Metal Impurities (As, Pb, and Cd) in Green Tea Camellia sinensis (L.) Samples (Infusions) Available in Poland
Source: Biol Trace Elem Res. 2023 May 2;202(1):387–96. doi: 10.1007/s12011-023-03665-5 (PMC10764562; doi:10.1007/s12011-023-03665-5)
Supplement: Supplementary file 1 — Supplementary file1 (DOCX 19 KB) [file 12011_2023_3665_MOESM1_ESM.docx]

**Supplementary Materials 1 (SM1)**

**Table 1.** The list of investigated Green Tea samples.

| **Code of Sample** | **Form of tea** | **The amount of raw material intended for a single Brewing Process, g** | **Time of the**  **Brewing Process (Brew Time), Minutes** | **Country of Origin** | **EAN** |
| --- | --- | --- | --- | --- | --- |
| GT1 | Tea bag | 1.5 | 1-3 | Sri Lanka | 5901086000333 |
| GT2 | Tea bag | 1.5 | 3 | China | 5900175401532 |
| GT3 | Leaf tea | 2.0 | 2-3 | China | 5900738004101 |
| GT4 | Leaf tea | 2.0 | 3 | Sri Lanka | 5901483051129 |
| GT5 | Leaf tea | 4-5 | 3 | China | 5907732943986 |
| GT6 | Leaf tea | 3.0 | 4 | China | 5900956700410 |
| GT7 | Tea bag | 1.5 | 1-3 | China | 5906881826072 |
| GT8 | Tea bag | 2 | 2-3 | Sri Lanka | 4796004230449 |
| GT9 | Tea bag | 2 | 3-4 | China | 5900956006782 |
| GT10 | Tea bag | 1.5 | 1-3 | Sri Lanka | 4791038950158 |
| GT11 | Leaf tea | 2.0 | 3-5 | Sri Lanka | 5900396000736 |
| GT12 | Tea bag | 1.75 | 2-3 | China | 20321154 |
