# Supplementary material for: The Control and Comprehensive Safety Assessment of Heavy Metal Impurities (As, Pb, and Cd) in Green Tea Camellia sinensis (L.) Samples (Infusions) Available in Poland
Source: Biol Trace Elem Res. 2023 May 2;202(1):387–96. doi: 10.1007/s12011-023-03665-5 (PMC10764562; doi:10.1007/s12011-023-03665-5)
Supplement: Supplementary file 2 — Supplementary file2 (DOCX 15 KB) [file 12011_2023_3665_MOESM2_ESM.docx]

**Supplementary Materials 2 (SM2)**

*The analytical calibration strategy and quality control*

To ensure appropriate quality standards, the properly developed calibration strategy and quality control approach were made. The quantitative analysis of elements in investigated infusion samples were made applying analytical calibration approach (calibration curves) obtained by diluting a stock standard of the elements studied (1:100 – 1:10000). The working solutions (*n* = 5) with concentrations for Pb and Cd: 0.0; 1.0; 2.0; 5.0; 10.0 µg/L. For As: 0.0; 10.0; 20.0; 50.0; 100.0 µg/L were prepared and applied for the analytical calibration procedure.

The obtained values of correlation coefficient (0.995 < R < 0.999) indicated that the analysis was precise and accurate. The summary of analytical calibration strategy and quality control results are shown in Table S1.

**Table S1.** The summary of analytical calibration strategy and quality control results.

| Analyte | calibration function | | R | recovery, % |
| --- | --- | --- | --- | --- |
|  | sigma A | slope |  |  |
| As | 2.31374 | 1404.5 | 0.99996 | 98 ± 0.9 |
| Pb | 66.356 | 6901.3 | 0.99961 | 98 ± 0.7 |
| Cd | 5.87525 | 1204.49 | 0.99996 | 99 ± 0.5 |

For quality control, we obtained values for recoveries: 98.7%, 98.6 and 98.5% for As, Pb and Cd, respectively. The calculated limits of quantification (LOQs) were 0.46 µg/L 0.39 µg/L and 0.053 µg/L for As, Pb and Cd, respectively. The calculated limits of detection (LODs) were 0.14 µg/L, 0.12 µg/L and 0.016 µg/L for As, Pb and Cd, respectively.
