# Supplementary material for: The Control and Comprehensive Safety Assessment of Heavy Metal Impurities (As, Pb, and Cd) in Green Tea Camellia sinensis (L.) Samples (Infusions) Available in Poland
Source: Biol Trace Elem Res. 2023 May 2;202(1):387–96. doi: 10.1007/s12011-023-03665-5 (PMC10764562; doi:10.1007/s12011-023-03665-5)
Supplement: Supplementary file 3 — Supplementary file3 (DOCX 15 KB) [file 12011_2023_3665_MOESM3_ESM.docx]

**Supplementary Materials 3 (SM3)**

**Table SM1.** The ratios (%) of the obtained values for heavy metals (As, Pb and Cd) of weekly intake (µg/kg bw/week) to the established PTWI.

| Green tea sample (infusion) | The ratios (%) of the obtained values of weekly intake (µg/kg bw/week)  to the PTWI for each element | | |
| --- | --- | --- | --- |
|  | As | Pb | Cd |
| GT1 | 0.13-0.43 | 0.21-0.71 | 0.17-0.58 |
| GT2 | 0.22-0.72 | 0.41-1.36 | 0.30-0.99 |
| GT3 | 0.23-0.78 | 0.36-1.19 | 0.28-0.93 |
| GT4 | 0.16-0.53 | 0.30-0.99 | 0.24-0.79 |
| GT5 | 0.09-0.30 | 0.17-0.57 | 0.11-0.38 |
| GT6 | 0.14-0.47 | 0.17-0.55 | 0.16-0.55 |
| GT7 | 0.17-0.58 | 0.20-0.68 | 0.17-0.56 |
| GT8 | 0.03-0.10 | 0.11-0.36 | 0.12-0.40 |
| GT9 | 0.08-0.27 | 0.17-0.56 | 0.16-0.53 |
| GT10 | 0.05-0.17 | 0.09-0.31 | 0.11-0.36 |
| GT11 | 0.16-0.52 | 0.18-0.61 | 0.18-0.59 |
| GT12 | 0.10-0.34 | 0.20-0.66 | 0.21-0.69 |
